# The Well-Being of Primary School Teachers during COVID-19

**DOI:** 10.3390/ijerph191811177

**Published:** 2022-09-06

**Authors:** Hjordis Sigursteinsdottir, Gudbjorg Linda Rafnsdottir

**Affiliations:** 1 Faculty of Business Administration, University of Akureyri, Nordurslod 2, 600 Akureyri, Iceland; 2 Faculty of Social and Human Science, University of Iceland, Saemundargotu 2, 102 Reykjavik, Iceland

**Keywords:** perceived stress, mental health, physical health, primary school teachers

## Abstract

This study examines the self-rated health and well-being of Icelandic teachers just before and over a year after COVID-19 first appeared. We ask, what was the stress level in 2021 compared to 2019 and the impact of mental and physical health and health symptoms on perceived stress? Were there any changes in self-assessed mental and physical health? Were there any changes in self-assessed mental and physical health symptoms? The study is based on an online survey conducted in 2019 and 2021. A total of 920 primary school teachers answered the questionnaire in part or in full, after three reminders. The main findings show increased stress, worsening mental and physical health, and increasing mental and physical symptoms in 2021 compared to 2019. The results also show a higher percentage of women than men reporting high stress, with women scoring higher on the PSS scale, but the gender patterns for mental and physical health are less clear. The results show that the COVID-19 pandemic had negative consequences on the health and well-being of the teachers. The study demonstrates the importance of school authorities keeping an exceptionally watchful eye on the welfare and well-being of teachers in the wake of the COVID-19 pandemic.

## 1. Introduction

In numerous studies, teaching has been identified as a profession with high stress levels (see e.g., [1,2,3,4]). In line with that, Kagwe, Ngisi, and Mutisya [5] point out that teachers are expected to fill many roles in their daily tasks in addition to teaching, such as being information providers, social workers, disciplinarians, planners, evaluators, facilitators, and role models. Increasingly, teachers need to meet the requirements of parents, colleagues, and stakeholders, such as local governments, sometimes without having their achievements appreciated [6,7]. Due to the workload, these many and sometimes contradictory roles can lead to physical and mental health problems [8]. It is known that chronic exposure to stressful work-related conditions can lead to several mental disorders, such as anxiety and depression, with increases in absenteeism and declines in teachers’ performance as consequences [9]. Stressful work-related conditions can also lead to physical health problems, such as heart disease and musculoskeletal and gastrointestinal symptoms [10,11]. For these reasons, research on the occupational health and well-being of teachers, like we are presenting here, is important. Teachers’ health is not only important to themselves. Due to their close connections to students, teachers’ well-being can affect their students’ performance and the learning process [9].

Unfortunately, economic crises regularly plague societies. Studying the health and well-being of key professions in society, such as teachers, during a period of economic crisis is, therefore, essential. Marmot and Bell [12] predicted that the economic crisis that swept the world in the wake of the bank crisis in 2008 would likely have a negative effect on people’s health, no less than on the economy. Gonza and Burger [13] are among those who showed that their prediction was correct, as that crisis had a negative effect on public health in the 36 countries they studied during that crisis. In that same vein, Sigursteinsdottir and Rafnsdottir [14] showed that health and well-being among employees within the education and care sectors in Iceland worsened in the wake of the economic crisis in 2008 and that these adverse effects became more robust over time. The same applies to harassment and bullying [15] and threats and physical violence [16]. In these studies, no significant difference was found between women and men. However, Sigursteinsdottir and Rafnsdottir’s [14] study showed that young teachers were likelier to become sick in the wake of the 2008 economic crisis than older ones.

The most recent global economic crisis was the one that followed the COVID-19 pandemic. Studying teachers in Spain, García et al. [17] showed that the implications of the confinement resulting from the COVID-19 pandemic have been unpredictable and diverse. Our goal in this article is to improve this knowledge by introducing a study on the health and well-being of teachers before and during the COVID-19 pandemic.

### COVID-19 Pandemic and the Well-Being of Teachers

The COVID-19 pandemic affected various other factors that can be directly attributed to the virus, such as gender equality [18], work–life balance [19] and poverty [20]. Although economic crises often have a broader impact on the labour market predominantly occupied by men, the COVID-19 pandemic is different, as it had an enormous impact on the ‘in-person jobs’ often dominated by women. This applies to teaching, as teachers are less likely than many other employees to be able to protect themselves by maintaining a social distance from other individuals, such as students. Consequently, people working in these areas tend to be less protected from getting sick. Due to the nature of the work, teachers have frequent contact with young families, who, according to Spinelli et al. [21], were at increased risk of developing psychological problems during the pandemic because of stressful experiences, such as lockdowns. MacIntyre et al. [22] also point out possible catalytic effects, such as fear of contagion and uncertainty about the duration of the pandemic and its possible impact on the economic situation. As a result, psychophysical manifestations of distress might emerge, with severe consequences, depending on the case. In a study by Rubilar and Oros [23], among teachers of all educational levels in Argentina, more than 60% of the educators reported moderately high and high levels of stress. The predominant stressors were uncertainty about the consequences of the pandemic, work overload, and an inadequate working environment. The results also revealed that the more stress the teachers experienced, the higher the manifestation of unwanted psychophysical symptoms and physical symptoms. To measure the prevalence of anxiety, depression, and stress among teachers during the COVID-19 pandemic, Ozamiz-Extebarria et al. [24] conducted an online questionnaire among teachers in the Basque community in Spain when schools were reopened. They showed that the teachers reported levels of anxiety (17%), depression (19%), and stress (30%), and there were no significant differences regarding gender and age in any of the symptoms. According to Klapproth et al. [25], teachers in German schools experienced moderately high levels of stress on average during COVID-19. What consequences this has had on teachers is essential to study. The research presented here will add to this knowledge.

The study presented here was conducted in Iceland; the virus was confirmed to have reached the country in February 2020. The infection rate was one of the highest in the world (possibly due to extensive screening), and the pandemic influenced the operations of schools, as in many other countries. However, around 92% of primary schools remained open, albeit with some restrictions to minimise the risk of spreading the pandemic among schoolchildren. These restrictions included grouping students and organising their attendance in school at different times or days. Almost all schools started distance teaching in one way or another to support students’ homework [26]. They had to change the curriculum considerably, so teachers spent more time than before on teaching and planning. They also spent additional time in the dissemination of information and communication with the students’ families [26]. While many of the parents worked from home to protect their health, most teachers were in school taking care of the children. At the same time, some teachers expressed that they were thrilled to finally be seen as ‘essential workers’, placed on the frontline alongside healthcare professionals [27].

That different groups of teachers experienced the situation during the pandemic differently in Iceland is in line with Mondragon et al. [28], who showed that Spanish teachers’ self-reported quality of life during the pandemic differed according to their personal and professional characteristics. Lizana and Lera [29] and García et al. [17] further showed that female Spanish teachers perceived work–family interactions during the pandemic more negatively than men. Lizana and Lera [29] identified an increased risk of symptoms of anxiety and stress among teachers who did not experience good work–family interactions. Gender and age seem to matter, as depression symptoms were higher among female than male teachers during the pandemic, and teachers under 35 years old had a higher risk of suffering from the three symptoms measured: depression, anxiety, and stress. However, there are not only differences between groups of people but also between schools. Garcia et al. [17] showed that Spanish teachers in private and subsidised schools received more work-related support during the pandemic than those in public schools. This is in line with Lizana and Lera’s [29] results from Chile, which showed that teaching in private educational establishments was a protective factor against anxiety symptoms.

The studies mentioned above were all conducted after the COVID-19 pandemic took place. The strength of our study presented here, and what separates it from the above-mentioned studies, is that the study uses data from a longitudinal panel study conducted among teachers both before and after the pandemic—more concretely, one year before and one year after the virus reached Iceland. Therefore, the aim of the study was to measure what changes, if any, occurred in self-rated occupational health among teachers between the two time points of the study. More concretely, our research questions are as follows:

What was the stress level in 2021 compared to 2019 and the impact of mental and physical health and health symptoms on perceived stress?

Were there any changes in self-assessed mental and physical health?

Were there any changes in self-assessed mental and physical health symptoms?

We analysed the data with regard to gender, age, marital and family status, number of years in teaching, and employment rate.

## 2. Materials and Methods

Data used in this study were from the longitudinal panel study, called *Health and well-being of Icelandic municipal employees,* conducted between 2010 and 2021. The data used here were collected using pretested questionnaires sent electronically to the participants in 2019 and 2021. The participants came from 12 of the 64 municipalities in Iceland. The ethics committee of the University of Iceland approved the research.

### 2.1. Procedure and Participants

The participants in this study were 920 primary school teachers in 12 municipalities in Iceland who participated at time 1 (2019) and 2 (2021). Public schools are run by the municipalities, which is by far the most common form of school in Iceland.

In 2019, a total of 2125 teachers (73%) completed the questionnaire in full or in part; 920 also answered the questionnaire in 2021. Table 1 show the characteristics of study participants. Most participants were women (82.5%), reflecting the gender composition of primary schools in Iceland. According to the Icelandic Teachers’ Union [30], 85.2% of primary school teachers are women. The average age of the teachers was 46.8 years, which was slightly higher among women, at 46.9 years, compared to 46.2 years for men. Proportionately, most teachers (36.0%) were in the age range 41–50 years, and 25.0% were in the age range 51–60. In most cases (77.4%), the teachers were married or cohabiting and in a household with children (69.3%). Most of the teachers were in full-time positions (79.8%), and 53.6% had been working as teachers for 11 years or longer.

### 2.2. Measures

The 10-item Perceived Stress Scale (PSS-10) was used to measure the respondents’ perceptions of stress [31]. The purpose of the PSS is to assess how much stress a person experienced in the last month. The respondent estimates how often life seemed unpredictable, uncontrollable, and/or overloaded. The 5-point Likert scale ranging from 0 (never) to 4 (very often) includes six positive and four negative items to measure psychological distress (e.g., ’In the last month, how often have you felt that you were unable to control the important things in your life?’). The total score on the scale ranges from 0 to 40 (with higher scores representing higher stress levels [31]). A mean score of ≥ 13 is categorised as ‘average stress’, and ≥ 20 is categorised as ‘high stress’. The intrinsic validity of the PSS-10 scale has been measured at 0.85, according to the alpha coefficient [31,32]. In this study, the alpha value was measured as 0.86.

Mental and physical health was measured with three questions: (1) ‘Is your mental health better or worse than it was 12 months ago?’ and (2) ‘Is your physical health better or worse than it was 12 months ago?’ There were five response options for those two questions: 1 = much better than 12 months ago; 2 = somewhat better than 12 months ago; 3 = the same as 12 months ago; 4 = somewhat worse than 12 months ago; and 5 = much worse than 12 months ago. Regarding health symptoms, the question was, (3) ‘Do any of the following symptoms interfere with your daily life: lack of strength, myositis, pain in the back/shoulders, frequent headaches, abdominal pain, dyspnoea, sleep disturbances, extreme worry, anxiety, sadness, arrhythmias, hypertension, colon spasms, and colds/fevers?’ There was a choice of four responses: 1 = Yes, last month; 2 = Yes, last three months; (3) Yes, last six months; and 4 = No, never. In addition, there were questions about gender (male/female), age (year of birth), marital status (married or cohabiting/single, divorced, widowed), parental status (with children/no children), working experience as a teacher (less than a year/1–5/6–10/11–20/21 years and longer), and employment rate (full-time/part-time).

### 2.3. Statistical Processing

The results are presented as numbers, percentages, averages, standard deviations, odds ratios, and associated *p*-values. A paired-samples t-test was conducted to evaluate the stress level in 2021 compared to 2019. An independent-samples t-test was conducted to explore the difference between PSS score and gender, marital status, parental status, and employment rate in 2019 and 2021. A one-way ANOVA was conducted to explore the difference between PSS score and age and working experience as a teacher in 2019 and 2021. McNemar’s test was used to evaluate if there were any changes in self-assessed mental and physical health and mental and physical health symptoms between 2019 and 2021. Generalised estimating equations (GEEs) were used to evaluate the impact of mental and physical health and mental and physical symptoms, gender, age, marital status, parental status, work experience as a teacher, and a full-time or part-time position on perceived stress over time. In Model 1, the main effects of mental and physical health and mental and physical symptoms were examined. In Model 2, gender, age, marital status, and parental status were added to Model 1. In Model 3, teacher experience and full-time or part-time positions were added to Model 2. This shows an interaction between time and mental symptoms, time and physical symptoms, time and worse mental health, and time and worse physical health. We obtained the odds ratio (OR) and associated *p*-value for each predictor in the GEE models. Statistical tests were conducted at a 5% level of significance, and data analyses were conducted using SPSS version 22.0. The number of participants varied slightly in the analysis because of some missing values for the predictor variables.

## 3. Results

### 3.1. Stress Level in 2019 and 2021 and the Impact of Mental and Physical Health and Health Symptoms on Perceived Stress

The results revealed an increased mean score on the PSS, from 13.5 to 15.4 (*t*_(815)_ = −7.59; *p* = 0.001), indicating higher stress levels in 2021 than in 2019. In 2019, 18.8% of the teachers scored 20 or higher on the PSS scale, showing high stress, according to Cohen (1983), and in 2021, the proportion was higher, at 28.0%. Table 2 shows a summary of the mean score for the PSS in 2019 and 2021 by gender, age, marital status, parental status, work experience, and working full-time or part-time. An exact McNemar’s test determined that the difference in the proportion of high stress in 2019 and 2021 was statistically significant (*p* = 0.001). The results also show that the average score for stress turned out to be significantly higher in 2021 than in 2019 in all groups examined (*p* < 0.05), except for teachers aged 30 and younger and teachers who worked for less than a year for the municipality (*p* > 0.05).

The results showed that the mean score for the PSS was higher for women than men in 2019 (*t*_(845)_ = −2.51; *p* = 0.012) and in 2021 (*t*_(863)_ = −2.37; *p* = 0.018). There was also a significant difference in mean PSS score by age in 2019 (*F*_(4, 842)_ = 7.97; *p* = 0.001) and in 2021 (*F*_(4, 860)_ = 17.53; *p* = 0.001). The post hoc tests revealed a lower mean score on the PSS by the 51–60 age group than the other age groups in 2019 and 2021. There was no difference in the mean score for the PSS by marital status in 2019, but, in 2021, the mean score for the PSS was higher among teachers living alone than among teachers who were married or cohabiting (*t*_(863)_ = −2.46; *p* = 0.014). Furthermore, the mean score for the PSS was higher among teachers living with children both in 2019 (*t*_(845)_ = −3.30; *p* = 0.014) and in 2021 (*t*_(840)_ = −6.25; *p* = 0.001). However, the results revealed no differences in the mean score for PSS depending on whether the teachers were in full-time employment or part-time employment (*p* > 0.005).

Table 3 outlines the results of the GEE analysis predicting the likelihood of perceived stress over time. In Model 1, worse mental health, worse physical health, mental symptoms, and physical symptoms were all significantly associated with perceived stress (*p* < 0.05), but the time point of the study was not (*p* > 0.05). These results indicate that teachers with worse mental health (OR = 5.649), teachers with measured mental symptoms (OR = 4.509), teachers with worse physical health (OR = 0.671), and teachers with physical symptoms (1.602) were likelier to score higher on the PSS scale, controlling for other factors in the model. Even after adding gender, age, marital status, and parental status (Model 2) plus years in teaching and full-time or part-time positions (Model 3) to the model, worse mental health (OR = 5.380), worse physical health (OR = 0.743), mental symptoms (OR = 4.414), and physical symptoms (OR = 1.340) remained significant factors (controlling for other factors in the model).

The goodness of fit of the models in Table 3 improved by adding gender, age, marital status, parental status, years in teaching, and full-time or part-time positions (Quasi-likelihood under the Independence Model Criterion (QIC) and Corrected Quasi-likelihood under the Independence Model Criterion (QICC) became smaller). Model 4 shows a significant interaction between the time point of the study and mental symptoms (OR = 1.639), indicating that the influence of time was greater with teachers who reported mental symptoms.

### 3.2. Changes in Self-Assessed Mental and Physical Health

Table 4 shows the proportion of respondents for whom mental and physical health deteriorated in the last 12 months. The results revealed that 9.6% of the teachers reported much worse and somewhat worse mental health in 2019, but the ratio rose to 31.1% in 2021 (*p* < 0.001). Furthermore, the results show a significantly higher proportion of worse mental health in 2021 than in 2019 for all groups examined (*p* < 0.05), except for teachers 30 years old and younger, teachers older than 60 years, and teachers who had worked for less than a year as a teacher (*p* > 0.05).

The proportion of those who reported much worse and somewhat worse physical health rose from 16.5% in 2019 to 26.5% in 2021 (*p* < 0.001). There was a significantly higher proportion of worse physical health in 2021 than in 2019 for most groups examined (*p* < 0.05), but not for teachers 61 years and older, teachers not living with children, teachers who had worked as teachers for 11 years or longer, and teachers in part-time employment (*p* > 0.05).

### 3.3. Changes in Self-Assessed Mental and Physical Health Symptoms

Table 5 shows the proportion of teachers with mental and physical health symptoms that interfered with their daily lives in the last 12 months. The most common mental symptom reported was sleep disturbances, as 50.2% and 55.5% of the teachers reported that sleep disturbances had interfered with their daily lives in the last 12 months in 2019 and in 2021, respectively (*p* > 0.05). Anxiety, extreme worry, and sadness increased statistically between the time points of the study (*p* < 0.05). The biggest change over time was in sadness, the incidence of which rose from 32.0% in 2019 to 47.9% in 2021. The most common physical symptom reported was pain in the back/shoulder and myositis; however, the proportion of teachers who reported that pain in the back/shoulder had interfered with their daily lives in the last 12 months almost stayed the same in 2019 and 2021, but the proportion of myositis decreased between 2019 and 2021 (*p* > 0.05). Five out of ten physical health symptoms increased statistically between the two time points of the study: lack of strength, frequent headaches, abdominal pain, dyspnoea, and arrhythmias (*p* < 0.05). The biggest change over time was in frequent headaches, the incidence of which rose from 43.7% in 2019 to 58.0% in 2021.

Of those teachers who had physical symptoms in 2021, 12.4% said they were due to COVID-19, as did 15.0% of those who had mental symptoms.

## 4. Discussion

The aim of this research was to study possible changes in self-rated health and well-being among primary school teachers one year before the pandemic (2019) and two years later (2021), when the pandemic had lasted for over a year. We compared possible changes in stress levels between the two points of the study and the impact of mental and physical health and health symptoms on perceived stress. Furthermore, we determined whether there were any changes in mental and physical health and health symptoms. We considered a possible link between health and well-being outcomes and gender, age, marital and family status, years in teaching, and employment rate.

In general, the results show increased stress, worsening mental and physical health, and increasing mental and physical symptoms in 2021 compared to 2019. Worse mental health and mental health symptoms were the strongest predictor of high stress levels. The interaction between the time point of the study and mental health symptoms indicated that the influence of time was greater for teachers who reported mental symptoms than others. In other words, the COVID-19 pandemic has had a negative correlation with these health and well-being factors, indicating the deteriorating health and well-being of the teachers. Therefore, efforts should be made both to improve the health and well-being of employed teachers and to organise preventive measures to improve the working arrangements and ensure the well-being of future teachers. Now, we will discuss the results in more details.

The proportional stress level increased by around 10% in 2021 compared to 2019, and the result revealed that 28% of the teachers were categorised with high stress. This is in line with Rubilar and Oros’ [23] results, where about 60% of teachers of all educational levels in Argentina were categorised with levels of stress between moderately high and high, and with Klapproth et al.’s [25] results, where German teachers experienced moderately high levels of stress on average. In these studies, most teachers believed the reason for the stress to be the lack of technological equipment, Internet connectivity, excessive workload, and students’ demotivation as internal and external obstacles that made distance educational work difficult. We cannot say for sure whether similar factors influenced the stress level of the teachers in our study or whether they probably experienced a catalytic effect of COVID-19, as discussed by McIntyre et al. (see [22]). However, a study by Jonsdottir [26] revealed changes in the daily tasks of teachers in Icelandic schools during the pandemic, which we argue possibly made the teachers’ stress levels higher in 2021 than 2019. In other words, insecurity and changing work arrangements during the pandemic may have been stressful for the teachers, but we intend to investigate this further.

In line with other studies (e.g., [17,29]), our results show that a higher percentage of women than men reported high stress, and women scored higher on the PSS scale, both before and during the pandemic. Nevertheless, the proportional increase between the two time points of the study was similar for both genders. Although the increase in stress is apparent between the two time points for all age groups, fewer teachers over 51 years old felt high stress compared to the younger ones, and the mean score was lower. Further, teachers with children were likelier to report stress than other participants, especially during the pandemic. In our study, we did not ask about work–life balance. However, it is worth mentioning that the reason for more stress among teachers with children than others could be that work–life balance was difficult for some groups, especially for young families who had to work at home, even when their children were also staying at home due to the reduction in school attendance [19]. During this time, and when teachers had to struggle with their own work and family lives, they also had to support their students’ families, who struggled with the same situation. Thus, due to the nature of the work, teachers have a great deal of contact with young families who, according to Spineli et al. [21], experienced increased stress during the pandemic. So, in addition to changes in the organisation of work due to the COVID-19 pandemic, younger teachers with children might have experienced increased stress and strain, not only because of stressful situations in their own work and families but also because of a spread effect from other families related to their students.

In our data, teachers who were not married or cohabiting were likelier to experience high stress during COVID-19, but no difference was found between the groups before the pandemic. The reason might be the well-known isolation that younger and single individuals experienced during the pandemic, due to severe restrictions on social activities.

In line with the stress, our data show changes in self-assessed mental health for the worse among the teachers between 2019 and 2021 in all groups examined, except for teachers 30 years old and younger, teachers 61 years old and older, and teachers who had worked for less than a year as a teacher. The trend was similar for physical health in most of the groups examined, both related to age and children in the home, where teachers not living with children felt better than others. The proportion of participants reporting much worse and somewhat worse mental health rose from 10% in 2019 to 31% in 2021. In line with MacIntyre et al. [22], deteriorating mental health could be due to a catalytic effect of COVID-19, or due to changed roles of the teachers. According to Jonsdottir [26], these many roles and even contradictory roles can lead to physical and mental health problems [8]. We aim to study this further.

The gender patterns for mental and physical health are less clear than regarding stress. We cannot explain with our data why the results show more differences between women and men in relation to stress than mental and physical health, but Sigursteinsdottir and Rafnsdottir [14] and Sigursteinsdottir, Jonsdottir, and Rafnsdottir [33] argue that increased job demands mean less control at work, and less social support universally affected both female and male teachers to the same extent in the wake of the economic crisis in 2008, which most likely explains the absence of gender differences in relation to work-related health and well-being in their data. That might apply to our data as well. However, neither gender differences nor age differences are observed among Spanish teachers regarding anxiety, depression, or stress during COVID-19 [24]. Regarding stress, there was no significant increase in reporting bad mental and physical health during COVID-19 in the oldest age group. As measuring mental symptoms is one indicator of health and well-being, we asked if there were any changes in self-assessed mental and physical health symptoms among the teachers between the time points of the study. Most teachers (more than 50%) reported sleep disturbances, the only mental symptom that did not increase significantly during COVID-19. About every other teacher reported anxiety, extreme worry, and sadness in 2021, which is a significant increase from 2019. When asking about physical symptoms, the proportion was significantly higher in 2021 for 6 symptoms out of 10 compared to 2019, and the biggest change over time was in frequent headaches, which also could be connected to stress. De Simone et al. [34] show that workload and attitude towards changes in the work organization have significant direct effects on physical symptoms and indirect effects on physical symptoms through job satisfaction. Rubilar and Oros [23] further show that manifestation is higher of unwanted psychophysical and physical symptoms where perceived stress is high.

The measured increase in stress among teachers in our study are of a great concern, as the results show that worse mental and physical health, as well as mental and physical symptoms, were all significantly associated with perceived stress, after controlling for other factors in the models. This shows the importance of doing everything possible to improve work-related well-being among teachers. In line with Lizanna and Lera [29], who show that teachers under 35 suffered more from mental illness during the COVID-19 pandemic than older age groups, and with Sigursteinsdóttir and Rafnsdóttir’s [14] research on health and well-being among employees in education and care in the wake of the 2008 economic crisis, our data show the importance of looking specifically at the situation of young teachers and those with children at home.

The strength of the study is that it is based on panel data, in which employees answered questionnaires both before and during the COVID-19 pandemic—more concretely, one year before and after the virus reached the country. Therefore, we can measure if and what kinds of changes in health and well-being took place among teachers during the pandemic. In addition, the response rate was high (73%). The fact that we are not working with a sample, as all the teachers received the survey, makes the data strong. It could be seen as a limitation that we are only working with self-measured health assessments, without confirmation by physicians. Nevertheless, self-reported health has proven to be successful as a predictor of individuals’ future health and can be an early warning signal of future sick leave [35]. Moreover, our next steps are to interview teachers and school administrators to shed further light on the answers we received from the panel data and research further why the COVID-19 pandemic has had a negative correlation with the measured health and well-being factors in this study.

## 5. Conclusions

The theoretical contribution of the research lies mainly in the increased knowledge it creates of the connection between the COVID-19 pandemic and the lack of well-being among teachers as well as the interaction between stress and mental and physical well-being. The main practical contribution should appeal to those in charge of occupational health and prevention in the schools and school authorities. Since the negative effects of the economic crisis in 2008 on teachers’ health prevailed for years, the study demonstrates the importance of school authorities keeping an exceptionally watchful eye on the welfare and well-being of teachers in the wake of the COVID-19 pandemic. As economic crises tend to come periodically, organisations should be aware of this and take active measures to ensure that the workplace is a healthy and secure place for every employee. This knowledge is also important for those who work with policy development regarding teachers’ occupational health and safety. Although our study was on primary teachers in schools operated by Icelandic municipalities, we believe that the main results touch on such a fundamental element in well-being at work that they can be applied to different workplaces and other countries as well.

## Figures and Tables

**Table 1:** Characteristics of study participants (N = 920).

	Frequency	Percent
**Gender**		
Female	759	82.5
Male	161	17.5
**Age (years)**		
<30	45	4.9
31–40	212	23.0
41–50	331	36.0
51–60	230	25.0
>61	102	11.1
**Marital status**		
Married or cohabiting	712	77.4
Single, divorced, widowed	208	22.6
**Parental status**		
No children	282	30.7
With children	638	69.3
**How long have you been working as a teacher?**		
Less than a year	61	6.7
1–5	222	24.4
6–10	140	15.4
11–20	326	35.8
>21	162	17.8
**Full-time or part-time?**		
Full-time	733	79.8
Part-time	186	20.2

**Table 2:** Summary of mean score on the PSS by gender, age, marital status, parental status, work experience, and working full-time or part-time in 2019 and 2021.

		2019			2021			*t*-test
	N	Mean	SD	High Stress	Mean	SD	High Stress	*p*-Value
**Total**	816	13.47	6.39	18.8%	15.40	6.68	28.0%	0.001
**Gender**								
Female	669	13.74	6.34	19.9%	15.63	6.77	28.9%	0.001
Male	147	12.27	6.54	13.8%	14.34	6.20	23.5%	0.003
**Age (years)**								
<30	41	16.20	6.86	35.7%	17.24	6.54	36.4%	0.351
31–40	193	14.57	6.33	25.1%	17.40	6.31	38.6%	0.001
41–50	296	13.75	6.29	19.5%	15.62	6.58	28.3%	0.001
51–60	203	11.67	6.31	11.0%	14.50	6.46	22.5%	0.001
>61	83	12.98	5.83	12.2%	11.22	6.34	12.0%	0.020
**Marital status**								
Married or cohabiting	624	13.49	6.45	18.9%	15.10	6.68	27.3%	0.001
Single, divorced, widowed	192	13.40	6.23	18.3%	16.38	6.61	30.3%	0.001
**Parental status**								
No children	245	12.25	6.46	14.1%	13.20	6.39	18.8%	0.028
With children	571	13.99	6.30	20.8%	16.34	6.59	31.9%	0.001
**Working experience as teacher (years)**								
Less than a year	54	14.50	6.47	14.3%	15.87	7.04	29.8%	0.288
1–5	195	13.93	6.56	21.9%	16.70	6.64	33.7%	0.001
6–10	125	13.51	6.77	24.3%	15.46	6.36	25.0%	0.004
11–20	287	13.74	6.23	18.9%	15.52	6.59	29.6%	0.001
>21	148	11.93	6.03	11.9%	13.27	6.62	19.1%	0.017
**Full-time or part-time?**								
Full-time	655	13.45	6.35	18.3%	15.37	6.49	28.0%	0.001
Part-time	161	15.51	6.60	20.7%	15.51	7.43	27.7%	0.001

**Table 3:** Generalised estimating equation analyses predicting the likelihood of perceived stress.

	Model 1	Model 2	Model 3	Model 4
	OR	OR	OR	OR
Intercept	8.593 ***	11.062 ***	10.879 ***	10.683 ***
Follow-up study in 2021	0.161	0.219	0.218	0.428
Worse mental health	5.649 ***	5.396 ***	5.380 ***	6.217 ***
Worse physical health	0.671 *	0.717 *	0.743 *	0.600 *
Mental symptoms	4.509 ***	4.419 ***	4.414 ***	3.698 ***
Physical symptoms	1.602 ***	1.285 **	1.340 **	1.889 **
Women		0.661 *	0.796 *	0.837 *
Age		−0.074 ***	−0.061 ***	−0.059 ***
Single, divorced, widowed		0.486	0.460	0.430 **
With children		1.004 **	0.974 **	0.962 **
Years in teaching			−0.290 *	−0.284 *
Full-time position			0.459	0.460
Time 2 * mental symptoms				1.639 **
Time 2 * physical symptoms				−1.291
Time 2 * worse mental health				−1.319
Time 2 * worse physical health				0.265
Goodness of fit (QIC)	44,085.61	42,341.09	42,042.44	41,768.89
Goodness of fit (QICC)	44,085.87	42,339.31	42,040.52	41,768.04

Note: *** *p* ≤ 0.001, ** *p* ≤ 0.01, * *p* ≤ 0.05.

**Table 4:** The proportion of teachers for whom mental and physical health deteriorated in the last 12 months.

	Much Worse and Somewhat Worse Mental Health			Much and Worse Somewhat Worse Physical Health		
	2019	2021	McNemar’s Test *p*	2019	2021	McNemar’s Test *p*
**Total**	9.6%	31.1%	0.001	16.5%	26.5%	0.001
**Gender**						
Female	9.5%	32.3%	0.001	17.7%	25.9%	0.001
Male	10.3%	25.5%	0.001	11.0%	29.3%	0.001
**Age (years)**						
<30	23.3%	40.9%	0.263	20.9%	47.7%	0.012
31–40	13.1%	34.8%	0.001	18.0%	28.4%	0.019
41–50	8.9%	35.1%	0.001	13.1%	25.4%	0.001
51–60	4.7%	26.9%	0.001	15.9%	26.4%	0.003
>61	9.7%	14.7%	0.481	23.7%	16.8%	0.405
**Marital status**						
Married or cohabiting	9.9%	30.9%	0.001	16.7%	25.3%	0.001
Single, divorced, widowed	8.6%	31.8%	0.001	15.7%	30.8%	0.001
**Parental status**						
No children	8.9%	21.9%	0.001	20.4%	24.9%	0.171
With children	9.9%	35.1%	0.001	14.8%	27,2%	0.001
**How long have you been working as teacher?**						
Less than a year	14.3%	29.3%	0.064	14.5%	34.5%	0.001
1–5	10.3%	34.1%	0.001	14.2%	28.4%	0.001
6–10	12.6%	31.3%	0.001	18.2%	28.2%	0.028
11–20	9.5%	32.7%	0.001	18.6%	24.7%	0.073
21>	4.6%	24.1%	0.001	15.0%	22.2%	0.081
**Full-time or part-time?**						
Full-time	9.6%	31.5%	0.001	15.8%	27.4%	0.001
Part-time	9.7%	29.4%	0.001	19.1%	23.2%	0.222

**Table 5:** The proportion of teachers with mental and physical health symptoms that interfered with their daily lives in the last 12 months.

	2019		2021		McNemar’s Test *p*
	N	%	N	%	
**Mental symptoms**					
Sleep disturbances	294	50.2	332	55.5	0.174
Anxiety	240	41.3	333	55.5	0.001
Extreme worry	219	37.8	300	50.4	0.001
Sadness	185	32.0	285	47.9	0.001
**Physical symptoms**					
Pain in the back/shoulders	398	68.0	411	68.7	0.893
Myositis	369	63.1	407	53.8	0.166
Lack of strength	321	50.3	389	61.0	0.001
Colds/fevers	281	48.6	267	44.9	0.267
Frequent headaches	254	43.7	346	58.0	0.001
Abdominal pain	126	21.8	194	32.6	0.001
Hypertension	103	17.7	113	19.0	0.282
Dyspnoea	97	16.8	159	26.6	0.001
Arrhythmias	96	16.6	135	22.8	0.011
Colon spasms	91	15.7	117	19.6	0.094

## Data Availability

Not applicable.
